# The Norwegian version of the QOLIBRI – a study of metric properties based on a 12 month follow-up of persons with traumatic brain injury

**DOI:** 10.1186/s12955-017-0589-9

**Published:** 2017-01-19

**Authors:** Helene Lundgaard Soberg, Cecilie Roe, Cathrine Brunborg, Nicole von Steinbüchel, Nada Andelic

**Affiliations:** 1 Department of Physical Medicine and Rehabilitation, Oslo University Hospital, Postbox 4950, Nydalen 0424 Oslo, Norway; 2 Oslo Centre for Biostatistics and Epidemiology, Research Support Services, Oslo University Hospital, Oslo, Norway; 3 Institute of Medical Psychology and Medical Sociology, University Medical Centre Göttingen, Göttingen, Germany

**Keywords:** Disability, Metric properties, Outcome measure, Quality of life, Traumatic brain injury, QOLIBRI, GOSE

## Abstract

**Background:**

Consequences after Traumatic brain injury (TBI) affect the injured person’s self-image and quality of life. The purpose was to assess the health related quality of life (HRQoL) at 12 months after a TBI in patients admitted to regional trauma centres, and to evaluate the metric properties of the Norwegian version of the Quality of Life After Brain Injury (QOLIBRI) questionnaire.

**Methods:**

Two hundred four patients with TBI of all severities were included. HRQoL at 12 months post-injury was measured by the QOLIBRI. It has a total scale and 6 subscales (satisfied with Cognition, Self, Daily Life and Autonomy and Social Relationships, and bothered by Emotions and Physical Problems). Demographic and injury related data were registered. Disability was registered by Glasgow Outcome Scale Extended (GOSE) and Rivermead Post-Concussion Questionnaire, and mental health by Hospital Anxiety and Depression Scale. Descriptive statistics, internal consistency by Cronbach’s alpha and Corrected Item-Total Correlations were calculated. Rasch analysis, Principal Component Analysis (PCA) and Structural Equation Modelling (SEM) were applied.

**Results:**

Mean age was 37.6 (SD 15.4) years; 72% were men, and 41% had higher education. Over 60% were severely injured. Mean Glasgow Coma Scale score was 9.3 (SD 4.5). According to the GOSE 5.9% had severe disability, 45.5% had moderate disability, and 48.5% had good recovery at 12 months post-injury. The QOLIBRI scales had a high internal consistency (α = 0.75–0.96), and only Physical Problems had an α < 0.85. In the Rasch analysis all subscales and their items fit the Rasch model, except for the depression item in the Emotion subscale. PCA and SEM analyses supported a six-factor structure in a second-order latent model. The QOLIBRI supports an underlying unidimensional HRQoL model. The SEM model fit statistics of the second-order model indicated a moderate fit to the observed data (CFI = 0.86, TLI = 0.85, RMSEA = 0.076, SRMR = 0.061, χ^2^ = 1315.76, df = 623, *p*-value < 0.001).

**Conclusion:**

The Norwegian QOLIBRI has favourable psychometric properties, but there were some weaknesses related to its measurement properties of the total score when tested on a TBI population where many had severe TBI, and many had good recovery.

## Background

Traumatic brain injury (TBI) can have physical, cognitive, emotional and behavioural consequences, and the resulting impairments can give activity limitations and participation restrictions which subsequently representing lifelong disabilities [1–9]. These disabilities affect the injured person’s self-image and their quality of life [4, 10, 11]. Functional recovery after TBI largely varies according to its severity [12–15].

Activity, social integration and participation are key outcomes and core domains of rehabilitation research and are also considered essential for an individual’s quality of life (QoL) [16, 17]. Generic QoL concepts incorporate a person’s subjective sense of well-being in terms of their physical, psychological, and social functioning and support, as well as their coping strategies, self-efficacy, and self-conception [18, 19]. The concept of health-related quality of life (HRQoL) focuses on the specific impact of health on an individual’s subjective functioning and well-being [11, 20]. HRQoL can be operationalized through assessments of physical, psychological (emotional and cognitive), social and daily life domains, and these assessments are predominantly self-reported [10].

Two types of measures are associated with the concept of HRQoL. Generic instruments can be used to compare HRQoL across disease conditions, but they may not capture the particular problems typically experienced by those with a specific condition, e.g., TBI. In contrast, disease-specific HRQoL instruments are targeted to a specific health condition and should only contain items that are relevant to a specific disease; these questionnaires can therefore be particularly relevant in clinical settings. Given this background, the Quality of Life after Brain Injury questionnaire (QOLIBRI), a disease-specific self-report measure assessing the HRQoL of people after TBI, was developed in an international multicentre study [11, 21, 22]. The QOLIBRI assesses six dimensions of HRQoL according to six subscales (satisfaction with Cognition, Self, Daily Life and Autonomy, and Social Relationships and feeling bothered by Emotions and Physical Problems). The QOLIBRI is applicable to people with TBI of all severities and at all time points after the injury [22]. However, to date, no studies have measured the HRQoL of people with TBI of all severities using the QOLIBRI at a specific time point after their injury.

The metric properties of the QOLIBRI have previously been investigated using classical and modern test theory in an international sample of 795 persons who had experienced a TBI [11, 21]. In that study, the individual scales showed good internal consistency, with Cronbach’s alphas ranging from 0.75 for Physical Problems to 0.89 for Cognition and Self and 0.95 for the total score [11]; this good internal consistency was maintained in a subsample of patients with lower cognitive performance. Furthermore, Rasch analyses of each subscale and of the total score confirmed that the items had a satisfactory fit with their respective subscales. However, the Social Relationships and Physical Problems subscales showed a poor fit with a unidimensional model and only moderately supported the unidimensionality of the total scale [11].

The QOLIBRI was originally translated into Norwegian in 2008 in accordance with recommended procedures [23]. However, the metric properties of the Norwegian version of the QOLIBRI have not yet been published. Additionally, its properties have not been tested in people with TBI of all severities at 12 months post-injury, when a rather stable life situation either at home or in a supported living environment can be expected [24].

The aim of this study was to assess HRQoL 12 months post-injury and to evaluate the metric properties of the Norwegian version of the QOLIBRI and its subscales in patients across the spectrum of TBI severity. In particular, we aimed to test the dimensionality of the scale and its ability to capture the extent of patients’ problems. We hypothesized that the QOLIBRI would have satisfactory metric properties at 12 months post-injury in individuals with TBI of all severities.

## Methods

### Design and study population

**Fig. 1:**
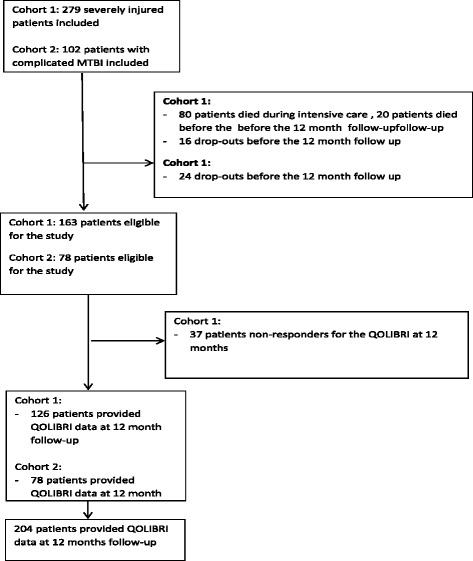
Flow chart of the patient inclusion

We conducted a cross-sectional study with 204 adult patients (age ≥16 years) post-TBI and measured HRQoL and post-injury functioning 12 months after their injury. The data were obtained from two Norwegian patient cohorts. Each cohort was participating in a large longitudinal research project assessing functioning and rehabilitation after sustaining a TBI. Cohort 1 consisted of 126 patients with severe TBI (STBI) according to the Glasgow Coma Scale (GCS) who were admitted to the Trauma Referral Centres of the four health regions of Norway in 2010. The inclusion criteria for this cohort were patients admitted to the hospital within 72 h post-injury who met the definition of STBI based on the lowest unsedated GCS score ≤ 8 in the first 24 h post-injury. Cohort 2 comprised 78 patients with mild TBI (MTBI) according to the GCS who were admitted to the neurosurgical department of Oslo University Hospital (OUH) from January 2010 through June 2011. The inclusion criteria for the MTBI cohort were admission to the OUH neurosurgical department, a GCS score of 13–15 and persisting post-concussion symptoms at an outpatient control at OUH 6–8 weeks post-injury. For both cohorts, the exclusion criteria were chronic subdural haematoma, pre-injury cognitive disability interfering with the assessment of outcome, severe psychiatric disease and drug abuse. Figure 1 shows a flow chart of the patient inclusion process.

### Measurements

All instruments were administered 12 months post-injury.

The QOLIBRI consists of 37 items that generate 6 subscales and a total score [11, 21]. A 5-point Likert scale ranging from 1 (not at all satisfied/bothered) to 5 (very satisfied/bothered) is used to assess satisfaction with Cognition, Self, Daily Life and Autonomy, and Social Relationships as well as feeling bothered by Emotions and Physical Problems [11, 25]. The QOLIBRI is scored according to an algorithm published by von Steinbüchel et al. [11]. Missing data were handled via imputation of the mean scale score if less than one-third of the responses in each subscale were missing. Raw scores were transformed into a score ranging from 0 (worst) to 100 (best) for the individual subscales, and a total scale score was also calculated.

The Rivermead Post-concussion Questionnaire (RPQ) is a self-report questionnaire measuring the severity of post-concussion symptoms following MTBI [26] and has previously been employed in populations with TBI of mixed severities [15]. This 16-item questionnaire assesses physical, emotional and cognitive symptoms after brain injury. Each item is scored from 0 (no problems) to 4 (severe problems), with a summary score ranging from 0 (best) to 64 (worst). An item score of 1 is rescored as 0 [26]. The RPQ was administered 12 months post-injury. Missing data were completed using the symptom profile reported in the patient’s medical record.

The Hospital Anxiety and Depression Scale (HADS) is a 14-item measure that has been validated for persons with TBI [27, 28]. The items of the HADS are rated on a scale ranging from 0 to 3. The score ranges from 0 to 42 (from best to worst) [27] and is used as an indicator of psychological distress. HADS scores between 15 and 18 are considered to indicate potentially clinically significant psychological distress requiring treatment, and people with scores of 19 and higher are considered to suffer from clinically significant psychological distress requiring treatment [27].

Global functioning and recovery were assessed using the Glasgow Outcome Scale-Extended (GOSE). The GOSE measures global functioning through a structured interview [29, 30]. According to the GOSE, patient status is categorized from 1 (dead) to 8 (upper good recovery). GOSE scores of 3–4 represent severe disability; scores of 5–6, moderate disability; and scores of 7–8, good recovery.

### Sociodemographic and injury-related information

The following sociodemographic variables were documented: sex, age at injury, and marital status. Education was categorized into two groups: low: fewer than 13 years of education; high: a university education. Employment status pre-injury and at 12 months post-injury was recorded and categorized as follows: being employed or a student; receiving sick leave, vocational or medical rehabilitation benefits, social security support or a disability pension; being unemployed; and being retired or a homemaker. Missing data for education were imputed based on the education required for the patient’s type of work.

The following injury-related data were assessed: GCS score [31], duration of post-traumatic amnesia (PTA), Abbreviated Injury Scale (AIS) score [32] and cause of injury. The lowest GCS score within the first 24 h post-injury, or at the site of injury in cases of pre-hospital intubation, was registered. The AIS grades the severity of each separate injury from 1 (minor injury) to 6 (maximal injury). The AIS score of the most severe brain injury, AIS-head, was recorded.

### Procedures and ethics

Baseline information was collected at the time of admission and from the Ulleval Trauma Registry at OUH [33]. At follow-up, the participants’ level of functioning and HRQoL were assessed using self-report questionnaires, clinical tests and structured patient interviews. Written informed consent for participation in the study was obtained, and the study was approved by the Regional Committee for Medical Research Ethics (#S-08378a (Cohort 1) and #171.08 (Cohort 2). None of the authors have any competing interests in the manuscript.

### Data analysis and statistics

Descriptive statistics were presented as means and standard deviation (SD) or medians and interquartile range (IQR). Correlations were analysed with Spearman’s ρ or Pearson’s r. Differences in continuous variables between groups were tested using independent sample t-tests or Mann-Whitney U tests.

The psychometric properties of the QOLIBRI were examined at the item level (A) and the scale level (B). The internal consistency of the scales was investigated, and item response theory/Rasch analysis was applied to test the fit of the items to the scale.

A) The responses to the items were checked to assess whether they were distributed throughout the entire range of possible options (1 to 5, “not at all” to “very”). In accordance with previous work by von Steinbüchel et al. [11], we used the WHOQOL group’s endorsement index (1998) to conduct the item frequency analysis. The distributions were examined for frequency problems to identify whether any two adjacent response categories had a sum of less than 10% of the total number of responses. Additionally, floor and ceiling effects were examined. A floor or ceiling effect was defined as >60% of the cases having the maximum or minimum score on a given QOLIBRI subscale [11].
  Each item was also assessed for skewness. Extreme skewness can reduce the probability that a scale will show strong correlations with other measures, thus reducing its precision and reliability. Extreme skewness can also indicate a deficit in the ability of different categories to appropriately discriminate responses to the target construct. Conventionally, items with skewness >1 are considered for removal; however, in accordance with the international study on the QOLIBRI, some moderately skewed items (1.0–1.3) were accepted to capture the range of disability [11].
B) The internal consistency of the QOLIBRI subscales and the total scale was examined using Cronbach’s alpha. Cronbach’s alpha ≥0.70 is considered acceptable, and values ≥0.90 are considered excellent [34]. The fit of the individual items within each subscale was tested by determining the correlation of each item with the total of the other items in its respective scale, and the corrected item-total correlations (CITCs) were calculated. In general, CITCs should be above 0.40 (WHOQOL group, [16]).

Additionally, a Rasch analysis of all items in the six subscales was performed to assess the fit of the items to the scale and to determine whether the ordinal scores fit the interval scaling requirements. No items were very skewed, and all categories had more than 10 responses. The ordering of response options and the item locations with standard error and fit (residual value with the Chi-square statistic and the corresponding probability value) were reported for each item. A standardized residual value greater than ± 2.5 and a probability value <0.001 are considered as misfits of an item [35, 36]. Bonferroni-corrected significance levels (0.05/number of items in the subscale) were used in the Rasch analysis. Targeting was evaluated by examining the hierarchical distribution of the items and their response levels and was compared to the distribution of the patients along the same metric scale. Rasch analyses were performed using RUMM2030 software.

Factor analysis was performed to study the structure of the Norwegian version of the QOLIBRI regarding the total scale and its subscales. Dimensionality was investigated using principle component analysis (PCA). We tested both a forced one-factor solution and a six-factor solution. Based on Kaiser’s criterion, we extracted all factors with eigenvalues >1 and applied an oblique rotation (promax method with an assumption of correlated scales). Finally, a confirmatory factor analysis (CFA) using structural equation modelling (SEM) was performed. The objective of CFA is to determine whether the data fit a hypothesized measurement model. This hypothesized model is based on previous analytic research and, in our study, on data from patients with mild-severe TBI [11, 21]. The analyses were conducted after imputation of missing values. To estimate the parameters, the maximum likelihood estimation procedure was applied in the CFA. The model fit of the QOLIBRI model was evaluated using Chi-square statistics, the Comparative Fit Index (CFI), the Tucker-Lewis Index (TLI), the Root Mean Square Error of Approximation (RMSEA), and the Standardized Root Mean Square Residual (SRMR). The fit statistics were interpreted based on the cut-off criteria proposed by Hu and Bentler [37]. Accordingly, a good fit of the model was indicated by non-significant Chi-square statistics, CFI and TLI > 0.95, SRMR < 0.07, and RMSEA < 0.06 using StataSE13.

## Results

### Descriptive statistics

The mean age of the 204 patients was 37.6 (SD 15.4) years; 72% were men, and 41% had a high level of education. Most (81%) individuals had been working or were students before they experienced the TBI. The demographic information is presented in Table 1. More than 60% of the patients were severely injured according to the GCS criteria. The patients had a mean GCS score of 9.3 (SD 4.5). According to the GOSE at 12 months post-injury, 5.9% had severe disability, 45.5% had moderate disability, and 48.5% had good recovery. The injury-related characteristics of the patients are presented in Table 2.

**Table 1:** Demographic information and post-injury functioning for the study population (*n* = 204)

Demographic information	
Age, mean (SD)	37.6 (15.4)
Gender (men)	147﻿ (72.1%)
Marital status	
- Married/living with a partner	98 (48.0%)
- Single/Divorced/Cohabitating^a^	105 (51.5%)
- Unknown	1 (0.5%)
Education	
- Low	121 (59.3%)
- High	83 (40.7%)
Pre-injury employment status	
- Employed/student	165 (80.9%)
- Sick-leave/vocational or medical rehabilitation/social security support/disability pension	17 (8.3%)
- Unemployed	7 (3.4%)
- Retired/homemaker	12 (5.9%)
- Unknown	3 (1.5%)
Employment status at 12 months	
- Employed/student	95 (75.4%)
- Sick-leave/vocational or medical rehabilitation/social security support/disability pension	69 (54.8%)
- Unemployed	6 (4.8%)
- Retired/homemaker	13 (10.3%)
- Unknown	3 (1.5%)
GOSE at 12 months (*n* = 202)	6.0 (IQR 6.0–8.0)
RPQ at 12 months *n* = (202)	14 (IQR 2.8–25)
HADS at 12 months (*n* = 201)	8 (IQR 3–13)

*GOSE* Glasgow Outcome Scale-Extended, *RPQ* Rivermead Post-concussion Questionnaire, *HADS* Hospital Anxiety and Depression Scale

^a^Includes apartment sharing

**Table 2:** Injury related data of the participants

Injury characteristics	
GCS (*n* = 204) mean (SD)	9.3 (4.5)
Median (IQR)	8 (6–15)
AIS-head mean (SD) (*n* = 202)	3.4 (1.4)
PTA	
MTBI (*n* = 78)	
<1 h	57 (73.1%)
1 h–< 24 h	16 (20.5%)
24 h–7 days	1 (1.3%)
Missing	4 (5.1%)
STBI (*n* = 126)	
<1 week	29 (23.0%)
1–2 weeks	17 (13.5%)
2–3 weeks	12 (9.5%)
3–4weeks	12 (9.5%)
>4 weeks	54 (42.9%)
Missing	2 (1.6%)
Injury mechanism (*n* = 204)	
- Traffic	88 (43.1)
- Fall	74 (36.3)
- Violence	19 (9.3)
- Other	23 (11.3)

*GCS* Glasgow Coma Scale, *AIS* Abbreviated Injury Scale, *PTA* Post-Traumatic Amnesia, *MTBI* Mild traumatic brain injury, *STBI* Severe traumatic brain injury

Scores displayed as mean (SD), median (IQR) or n (%)

At 12 months, 88% of the patients with STBI had returned home and 12% lived in institutional care facilities; whereas all participants with MTBI lived at home. 54.8% of the patients were still on sick leave or were receiving (vocational) rehabilitation benefits or a disability pension.

There were less than 5% missing responses for each QOLIBRI item. HADS scores were available for 98%, RPQ scores for 99% and GOSE scores for 99% of the patients (see Table 1).

The mean QOLIBRI score was 67.0 (SD 19.1) (see Table 3). There were no differences in the total QOLIBRI score between the cohorts, however, the patients with STBI reported 7.0 points better score on the Cognition subscale (*p* = 0.026) than reported by the MTBI patients. In addition, STBI patients showed a trend towards better higher score on the Self subscale by 5.9 points (*p* = 0.067) and on the Emotions subscale (*p* = 0.055) (data not shown).

**Table 3:** QOLIBRI mean scale scores and SDs

	Mean (SD)	α
Cognition	65.6 (21.9)	0.92
Self	62.3 (22.4)	0.91
Daily life and autonomy	66.3 (23.9)	0.90
Social relationships	69.4 (21.7)	0.85
Emotions	73.1 (24.4)	0.88
Physical problems	67.4 (22.9)	0.75
QOLIBRI Total	67.0 (19.1)	0.96 (all items)

Cronbach’s α for all data

According to the HADS results, 22% of the patients demonstrated symptoms of psychological distress at 12 months. Thirteen patients (6.5%) had mild to moderate symptom pressure, whereas 31 (15.5%) patients had scores ≥15 points, reflecting the presence of symptoms of anxiety and depression that required treatment (not shown in Table 1).

### Item characteristics and internal consistency

The reliability analysis (Table 4) showed that all CITCs within the respective subscales were greater than 0.40. The majority of items had CITCs greater than 0.60, and none had a CITC below 0.43. With respect to skewness, four items were skewed slightly above 1 (satisfaction with “ability to get out and about” and “relationship with members of your family” as well as being bothered by “feeling lonely” and “feeling angry or aggressive”). These items are clinically important and showed reasonable scale fits on the other parameters.

**Table 4:** Item descriptives and scale reliability analyses

	Item	Item descriptives Mean	SD	Percent missing	Skewness	CITC	Alpha if item removed
Cognition	Concentrate	3.34	1.19	0.5	- 0.48	0.788	0.897
	Express yourself	3.83	1.01	0.5	−0.55	0.785	0.898
	Remember	3.22	1.20	1.5	−0.31	0.676	0.911
	Plan and problem solve	3.80	1.09	1.0	−0.73	0.749	0.901
	Decisions	3.71	0.96	0	−0.47	0.731	0.904
	Find way	3.93	1.00	1.0	−0.81	0.673	0.909
	Speed of thinking	3.56	1.05	1.5	−0.55	0.803	0.896
Self	Energy	3.24	1.18	0.5	−0.35	0.697	0.895
	Control emotions	3.42	1.17	1.0	−0.49	0.737	0.890
	Motivation	3.46	1.12	0.5	−0.50	0.776	0.886
	Self-esteem	3.58	1.04	1.0	−0.39	0.638	0.900
	Way you look	3.70	1.16	0.5	−0.60	0.655	0.899
	Self-perception	3.56	0.99	1.5	−0.40	0.790	0.886
	Own future	3.47	1.16	1.5	−0.57	0.758	0.887
Daily life and autonomy	Independence	3.70	1.24	1.0	−0.67	0.762	0.876
	Get out and about	3.96	1.32	0.5	−1.07	0.663	0.887
	Domestic activities	3.78	1.11	1.5	−0.73	0.715	0.887
	Run personal finances	3.73	1.21	1.0	−0.78	0.616	0.893
	Participation in work	3.40	1.32	4.9	−0.53	0.693	0.884
	Social-leisure participation	3.37	1.24	4.9	−0.30	0.646	0.889
	In charge of life	3.61	1.20	2.0	−0.51	0.831	0.868
Social relationships	Affection towards others	3.96	1.08	0.5	−0.92	0.639	0.827
	Family	4.14	0.96	0.5	−1.13	0.632	0.830
	Friends	3.93	1.07	0.5	−0.77	0.723	0.812
	Partner	3.68	1.32	1.5	−0.72	0.690	0.818
	Sex life	3.26	1.37	2.5	−0.24	0.556	0.849
	Attitudes of others	3.71	1.01	2.0	−0.54	0.643	0.828
Emotions	Loneliness	4.15	1.09	1.5	−1.25	0.713	0.850
	Boredom	3.88	1.15	2.0	−0.93	0.671	0.859
	Anxiety	3.86	1.31	2.0	−0.83	0.752	0.840
	Depression	3.78	1.20	2.9	−0.71	0.836	0.819
	Anger/aggression	3.96	1.22	2.0	−1.08	0.581	0.880
Physical condition	Slowness/clumsy	3.92	1.21	2.0	−0.99	0.463	0.729
	Other injuries	3.54	1.43	1.5	−0.62	0.578	0.688
	Pain	3.63	1.39	1.5	−0.66	0.488	0.732
	See/hear	3.96	1.22	2.0	−0.99	0.426	0.741
	TBI effects	3.43	1.19	2.9	−0.32	0.663	0.662

*CITC* corrected item-total correlation, *SD* standard deviation

The internal consistency of each scale is shown in Table 3. Cronbach’s α ranged from 0.75 (Physical Problems) to 0.92 (Cognition) and was 0.96 for the total score. In this respect, the Norwegian QOLIBRI meets the necessary criteria to be applied in research and to provide acceptable and reliable assessments at the individual level.

### Response categories and targeting of the subscales

The results of the Rasch analysis are shown in Table 5. Concerning the Cognition subscale, all items revealed ordered response categories. The overall Chi-square statistic was 17.05 (df = 14, *p* = 0.25), indicating a fit to the Rasch model. All items in the Cognition subscale also fit the model. The mean person location was 1.27 (SD 1.95) indicating a higher level of satisfaction with cognitive function of the subjects than reflected by the subscale items.

**Table 5:** Measures of item difficulty and fit from Rasch analysis of each of the QOLIBRI scales

Item	Content	Location	SE	Standardized residual	*χ*2	Prob.
Cognition						
I 1	Concentrate	0.76	0.10	−1.01	2.33	0.31
I 2	Express yourself	−0.67	0.11	−1.27	1.79	0.41
I 3	Remember	0.95	0.10	2.36	1.58	0.45
I4	Plan and solve problems	−0.29	0.11	−0.15	0.76	0.69
I5	Decisions	−0.37	0.11	0.33	2.62	0.27
I6	Find a way about	−0.63	0.11	1.61	4.38	0.11
I7	Speed of thinking	0.24	0.11	−1.91	3.59	0.16
Self						
I1	Energy	0.54	0.10	1.14	1.81	0.41
I2	Motivation	0.18	0.10	−0.10	0.36	0.83
I3	Self-esteem	0.04	0.10	−1.23	3.15	0.21
I4	Way of look	0.26	0.10	1.69	1.79	0.41
I5	Achievements	0.37	0.10	1.21	5.38	0.07
I6	Self-perception	−0.28	0.11	−1.81	5.04	0.08
I7	Own future	0.16	0.10	−0.58	3.67	0.16
Daily life and Autonomy						
I1	Independence	−0.18	0.09	−0.93	2.28	0.32
I2	Get out and about	−0.11	0.13	−0.66	3.22	0.20
I3	Domestic activities	−0.42	0.10	0.17	1.95	0.38
I4	Run personal finances	−0.19	0.09	2.27	2.61	0.27
I5	Participation in work/education	−0.80	0.10	1.01	0.81	0.67
I6	Social and leisure activities	−0.22	0.09	1.88	2.74	0.25
I7	In charge own of life	−0.13	0.10	−3.05	6.51	0.04
Social Relationships						
I1	Affection to others	−0.33	0.09	0.34	5.49	0.06
I2	Family members	−0.73	0.10	−0.04	1.46	0.48
I3	Friends	−0.53	0.10	−0.98	8.25	0.02
I4	Partner	0.55	0.09	−0.74	0.82	0.66
I5	Sex life	1.15	0.10	2.12	3.31	0.19
I6	Attitudes of others	−0.12	0.10	0.62	1.26	0.53
Emotions						
I1	Loneliness	−0.20	0.10	−0.23	3.23	0.20
I2	Boring	0.34	0.10	0.72	0.64	0.73
I3	Anxiety	0.38	0.11	−0.57	4.82	0.09
I4	Depression	−0.85	0.17	−2.28	15.11	0.0005
I5	Anger	0.34	0.09	2.26	1.93	0.38
Physical Problems						
I1	Slowness/clumsy	−0.19	0.13	1.16	0.12	0.94
I2	Other injuries	0.37	0.12	0.20	1.00	0.60
I3	Pain	0.22	0.12	1.03	0.13	0.93
I4	See/hear	−0.34	0.12	1.02	0.15	0.93
I5	TBI effects	−0.07	0.09	−2.07	6.65	0.04

Bonferroni corrected significance level (0.05/number of items in the subscale)

*SE* standard error

Additionally, all items of the Self subscale were characterized by ordered response categories. The overall Chi-square statistic was 21.20 (df = 14, *p* = 0.10), indicating a fit of the Self subscale to the Rasch model. All items in the Self subscale also fit the model. The mean person location was 0.86 (SD 1.75) indicating a slightly higher level of satisfaction with self-perception of the subjects than reflected by the subscale items.

Items 2 and 5 of the Daily Life and Autonomy subscale revealed disordered thresholds and were rescored (1, 1, 2, 2, 3) and (1, 1, 2, 3, 4), respectively. After rescoring the items, the overall Chi-square statistic was 20.11 (df = 14, *p* = 0.13), indicating a fit of this subscale to the Rasch model. All items of the Daily Life and Autonomy subscale subscale also fit the model. The mean person location was 0.82 (SD 1.60), indicating that the subjects had a slightly higher level of daily activity than reflected by the subscale items.

Items 4 and 5 of the Social Relationships subscale demonstrated disordered thresholds and were rescored (1, 1, 2, 3, 4). The overall Chi-square statistic was 20.58 (df = 14, *p* = 0.06), indicating a fit of this subscale to the Rasch model. The mean person location was 1.03 (SD 1.50), implying that the subjects had a moderately higher level of social participation than the average of the subscale.

Disordered thresholds were found for items 3 and 4 of the Emotions subscale, and these items were therefore rescored (1, 2, 2, 3, 4) and (1, 2, 2, 3, 3), respectively. The overall Chi-square statistic was 25.72 (df = 10, *p* = 0.004), revealing a misfit of this subscale to the Rasch model, likely due to the misfit of item 4 (Table 5). The mean person location was 1.03 (SD 1.50), demonstrating that the subjects had a higher level of emotional function than reflected by the subscale items.

The disordered thresholds of items 1 through 4 on the Physical Problems subscale were rescored (1, 1, 2, 2, 3). The overall Chi-square statistic was 8.05 (df = 10, *p* = 0.62), indicating a fit of this subscale to the Rasch model. The mean person location was 0.55 (SD 1.33), suggesting that the subscale was targeting rather well between subjects and the subscale items.

### Exploratory factor analysis

The results of the two factor analyses (PCA) are shown in Table 6. A single-factor and a forced 6-factor solution were produced to compare the structure of the Norwegian version of the QOLIBRI to the results of the analysis of the QOLIBRI [11, 38]. The loadings on the single-factor solution for the total score showed an overall good fit in the first five scales, with the single factor solution as only four loadings were below 0.6 according to the First Principal Component scores, as shown in Table 6. Items in the Physical Problems scale had a weaker fit, with 4 factor loadings <0.6, although none of the items showed a poor fit (loadings <0.45).

**Table 6:** Principal component analysis of the Norwegian QOLIBRI items

Scale	Item	First principal component	Communality	Factor 1	Factor 2	Factor 3	Factor 4	Factor 5	Factor 6
Cognition									
	Concentrate	0.717	0.779	0.890					0.292
	Express yourself	0.764	0.729	0.820					
	Remember	0.685	0.602	0.569					0.350
	Plan and problem solve	0.736	0.678	0.739					
	Decisions	0.721	0.676	0.775					
	Find way	0.727	0.644	0.741					
	Speed of thinking	0.751	0.777	0.915					
Self									
	Energy	0.719	0.696	0.325	0.506				0.345
	Control emotions	0.726	0.670	0.355	0.613				
	Motivation	0.769	0.753		0.758				
	Self-esteem/Achievements	0.608	0.700		0.968				
	Way you look	0.758	0.627	0.475					
	Self-perception	0.776	0.747		0.708				
	Own future	0.763	0.692		0.680				
Daily life									
	Independence	0.663	0.759				0.814		
	Get out and about	0.593	0.675		0.291		0.736		
	Domestic activities	0.737	0.648	0.311			0.478		
	Basic personal needs	0.614	0.518				0.541		
	Run personal finances	0.671	0.598				0.583		
	Participation work/education	0.730	0.642		0.620				
	Social and leisure activities	0.813	0.726				0.441		
Social									
	Affection towards others	0.666	0.636		0.456			0.300	
	Family members	0.604	0.594	0.404				0.487	
	Friends	0.722	0.691	0.268				0.452	
	Partner	0.534	0.756					0.991	
	Sex life	0.549	0.651	−0.354				0.841	
	Attitudes of others	0.632	0.579					0.619	
Emotions									
	Loneliness	0.614	0.658			0.748			
	Boredom	0.542	0.662			0.806			
	Anxiety	0.609	0.729			0.827			
	Depression	0.707	0.808			0.798			
	Anger/aggression	0.560	0.595		−0.283	0.696			
Physical									
	Slow/clumsiness	0.490	0.648				0.751	−0.323	
	Other injuries	0.491	0.588						0.612
	Pain	0.566	0.504			0.376			0.373
	See/hear	0.479	0.567	0.264		−0.285			0.673
	TBI effects	0.653	0.695						0.634

Factor loadings > .25 are shown

The results of the 6-factor solution showed that most of the QOLIBRI scales loaded on the appropriate factors, i.e., their home subscale, and the PCA reproduced the overall structure of the international version of the QOLIBRI. Four items did not load on their home scale; “Achievements” loaded on Cognition instead of Self, “Social & leisure activities” loaded on Self instead of Daily Life, and “Slow/clumsiness” loaded on Daily Life instead of Physical Problems. In total, there were 16 cross-loadings (of the 37 items), and the social scale and the physical scale had the most cross-loadings (Table 6).

### Confirmatory factor analysis

**Fig. 2:**
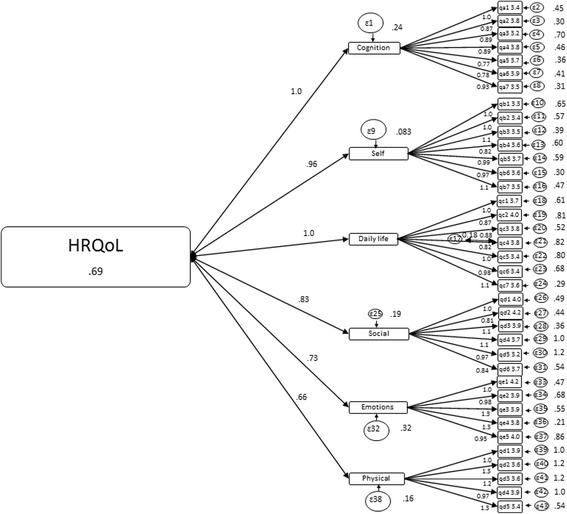
Structural equation model of the structure of the Norwegian QOLIBRI (standardized estimates)

SEM was used to assess the measurement structure of the QOLIBRI. Due to the high inter-correlations of the six latent factors (range of *r* = 0.55–0.85), a second-order HRQoL factor was included, as proposed by von Steinbüchel et al. [11]. Subsequently, the final model consisted of the six latent variables at the first-order level and HRQoL as a second-order latent variable (Fig. 2). The model fit statistics of the second-order model of an overall HRQoL model indicated a moderate fit to the observed data (CFI = 0.86, TLI = 0.85, RMSEA = 0.076, SRMR = 0.061, χ^2^ = 1315.76, df = 623, *p*-value < 0.001). The model meets the SRMR criterion where values < 0.08 are deemed acceptable in combination with TLI < 0.95, but not the RMSEA and the CFI criteria for satisfactory fit [37]. Thus, according to the test scores, the SEM analysis indicates that the HRQoL partially fits; however, the fit of the QOLIBRI total score, representing a common overall HRQoL model, fits less well.

## Discussion

The translation of the Norwegian version of the QOLIBRI was performed according to international standards and the requirements of its developers [11]. However, to administer the QOLIBRI in international studies, its metric properties have to be established as part of the cross-cultural development of the instrument. In the current study, we investigated the metric properties of the Norwegian version of the QOLIBRI with respect to its internal consistency, scale properties and factor structure. By testing all the subjects in this study at 12 months post-injury, potential ceiling effects in individuals in the spectrum of relatively mild to severe TBI, and other metric differences across the TBI subgroups could be evaluated.

The demographic characteristics of this study population are consistent with those of other studies, and the educational level was between those of the international (35%) and Finnish (50%) studies [11, 39]. The proportion of patients with STBI based on the GCS criteria in this study was also equivalent to that in other QOLIBRI studies; however, the current study contained a larger percentage of patients with good recovery (48.5%) than the international (28%) and Finnish studies (1.3%).

The patients were distributed throughout the entire spectrum of TBI severities. In this respect, the current study captured the variability in the HRQoL of TBI patients 12 months post-injury. However, the present results showed that STBI patients reported better cognitive functioning on the QOLIBRI subscale than the MTBI patients. The reduced awareness and longer rehabilitation periods with less exposure to job and other environmental demands for the STBI patients might be explanatory factors of the higher self-reported satisfaction with their cognitive functioning. Sasse et al. reported that a reduced awareness was associated with a higher satisfaction with cognitive functioning on the QOLIBRI [40]. Moreover, other studies have shown that people with reduced self-awareness report a higher HRQoL [41, 42]. Although QoL has been suggested to be overestimated due to reduced awareness, it is difficult to disregard on an individual’s perceived QoL. In addition, the social and emotional dimensions seem to be less overestimated than the physical dimension; this finding supports the validity of applying self-reported QoL also in the STBI group [43]. This might be explained by an overestimation of functioning by STBI patients than they have. Nonetheless, reliable HRQoL results have been obtained in other studies of patients with reduced cognitive functioning [44].

The internal consistency of the Norwegian QOLIBRI was generally satisfactory to excellent in this sample, in which all patients were severely injured or had a protracted course of recovery after MTBI. The lowest reliability according to Cronbach’s α was observed in the Physical Problems subscale, which covers a range of possible motor and sensory functions that can be expected to present differently within and between STBI and MTBI patients [45]. In accordance with the international study, the present result could be interpreted as indicating that the QOLIBRI captured the HRQoL of individuals along the entire spectrum of TBI severity [11].

The Rasch analysis indicated that the scoring categories adequately differentiated all items within the Cognition and Self subscales. For the other subscales, a couple of questions in each scale displayed disordered response thresholds for the scoring categories, with only three or four categories actually differentiating the level of the targeted item. Very few ordinal measurements fulfil scaling properties with distinct thresholds for the entire range of scoring options across all items [46]. Therefore, the results of the items of the QOLIBRI are deemed to be rather good. All subscales and their items fit the Rasch model, except for the depression item in the Emotion subscale, indicating a slight diversion in the anxiety and depression dimension that is also reflected in the HADS which calculates anxiety and depression separately [27, 28]. This finding supports the notion that the summary scores for each subscale provide valid measurements, with the exception of the Emotion subscale. The unidimensionality of QOLIBRI regarding emotions was documented in the study by von Steinbüchel et al. [11]. Rasch analyses are population-specific, and slight misfit to a single item is common [47, 48]. However, one could allocate for analysing anxiety and depression separately when applying the Emotional subscale of the QOLIBRI in the present population. The targeting of a scale is essential for measuring the problems of all subjects and the basis for determining the responsiveness of the measurement [49]. The subscales, with the exception of the Physical Problem subscale, targeted subjects with slightly more problems than experienced by the present population, which is not surprising given the one-year period of recovery after injury.

The PCA and SEM analyses of the Norwegian version of the QOLIBRI also supported the structure that was tested and reported by the QOLIBRI developers, with a six-factor structure in a second-order latent model [11]. The PCA showed that the QOLIBRI supports an underlying unidimensional HRQoL model. Our results are somewhat stronger than those reported in the international study [11]. In our study, the first four scales tapping on satisfaction with functioning, had a good fit, whereas the Social function scale in the international study showed a poorer fit. However, according to the SEM, our results indicate certain underlying weaknesses in the QOLIBRI model. The second order model showed moderate results, and did not quite obtain the fit criteria for a unidimensional latent HRQoL factor compared to Steinbüchel et al. [11]. However, our population included TBI patients of all severities at one point of time post-injury, whereas theirs comprised people with TBI of all severities studied at varying times after the injury. The postinjury experience might differ between these populations, which might influence the fit of the unidimensional factor structure. Similar problems were reported in the international study, where these problems may have been attributed to a large N; however, our study showed that there may be other underlying challenges as well in the QOLIBRI model.

As hypothesized, the Norwegian version of the QOLIBRI provides a subscale and total scale for different aspects of HRQoL, with acceptable psychometric properties. The six domains of the QOLIBRI covered a rich profile description, which is necessary in studies in which the changes in self-reported functioning within specific domains are of value, andthe QOLIBRI total score provides a measurement of the impact of interventions on the aggregated HRQoL.

In line with the international QOLIBRI study, the Norwegian version of the QOLIBRI demonstrated acceptable psychometric properties. In previous international studies on the development and metric properties of the QOLIBRI, most of the participating countries lacked a sufficient number of patients to confirm the factor structure in their respective languages [11]. However, a subsequent study from Finland found that the QOLIBRI was psychometrically sound [39], and a validation of the Australian version has also been published [25]. Both of these studies consisted of patients who were included in the international study. Hence, the current study is one of the first to test the factor structure of the QOLIBRI in a specific language independent from the international study. Given this context, our results support previous findings and strengthen the validity of the QOLIBRI.

The QOLIBRI was developed to capture HRQoL from the patient’s perspective. The current study showed that its metric properties were somewhat weaker on the two problem scales, pertaining to Emotions and Physical functioning. Although the latter scale captures functions that are clinically relevant, physical functioning might vary largely within the TBI population, often independently of TBI severity [50]. The items of the Physical functioning scale cover specific but very different functions, such as clumsiness, hearing/vision and pain. Although these are relevant functions, they may not capture the diversity of the spectrum of physical challenges experienced after sustaining a TBI [8]. Furthermore, the items in the QOLIBRI mainly assess respondents’ satisfaction and feelings of being bothered by emotional and psychosocial aspects following TBI, not their actual functioning per se. Hence, the assessment of HRQoL using the QOLIBRI can supplement other measures such as the GOSE in the rehabilitation of persons who have suffered a TBI.

This study has some limitations. The current study did not assess test-retest reliability because our data were from a clinical study and were not designed to be used in planned as a study of the metric properties of the QOLIBRI. However, the general results of our study do not deviate substantially from those of the international study, and we expect the test-retest reliability to be consistent between studies, as well. Furthermore, this study did not comprise results of detailed cognitive neuropsychological testing for all the patients. Only the STBI cohort underwent more comprehensive neuropsychological testing on a general basis, whereas a more extensive test battery was only administered to MTBI patients when indicated.

## Conclusions

In conclusion, the results of the analysis of the Norwegian version of the QOLIBRI indicate that it has favourable psychometric properties. However, there were some weaknesses related to the QOLIBRI measurement properties of the total score when tested on a population experiencing TBI, many of whom had very severe TBI and many of whom exhibited good recovery.

## Abbreviations

AIS: Abbreviated injury scale
CFI: Comparative fit index
GCS: Glasgow coma scale
GOSE: Glasgow outcome scale extended
HADS: Hospital anxiety and depression scale
HRQoL: Health related quality of life
IQR: Interquartile range
MTBI: Mild TBI
OUH: Oslo University Hospital
PCA: Principal componet analysis
PTA: Post-traumatic amnesia
QoL: Quality of life
QOLIBRI: Quality of life after brain injury
RMSEA: Root mean square error of approximation
RPQ: Rivermead post-concussion questionnaire
SD: Standard deviation
SEM: Structural equation modelling
SRMR: Standardized root mean square residual
STBI: Severe TBI
TBI: Traumatic brain injury
TLI: Tucker-Lewis Index
